# Signaling Molecules in Sulfur Mustard-Induced Cutaneous Injury

**Published:** 2007-11-27

**Authors:** Albert L. Ruff, James F. Dillman

**Affiliations:** US Army Medical Research Institute of Chemical Defense, Aberdeen Proving Ground, Maryland

## Abstract

**Objective:** Sulfur mustard (SM) is a potent alkylating agent that can induce severe cutaneous injury. Though much is known regarding the gross pathology of SM injury, the molecular and cellular basis for this pathology is not well understood. General cellular processes such as inflammation, DNA damage response, and apoptosis have been hypothesized to be involved in SM injury. However, the specific molecules, signaling pathways, and gene products involved in the pathogenesis of SM injury have not been elucidated. This review discusses the molecular mechanisms observed in *in vivo* and *in vitro* models of cutaneous SM injury. **Methods:** The historical literature on the clinical pathology of SM-induced cutaneous injury is summarized, and recent work elucidating molecular signaling pathways involved in SM toxicity is extensively reviewed. In addition, this review focuses the discussion of SM-induced molecular mechanisms on those that have been experimentally validated in models of SM injury. **Results:** Recent work has uncovered potential roles for a number of signaling molecules. In particular, molecules in inflammatory signaling, DNA damage response, apoptosis signaling, and calcium signaling have been implicated in SM injury. These include signaling molecules involved in inflammation (e.g. p38 MAP kinase), apoptosis (e.g. p53, NF-κ B, caspases, Fas), and cell stress responses (e.g. calcium, calmodulin). **Conclusions:** Many of the molecules and mechanisms implicated in SM injury are now being experimentally validated. Critical questions are proposed that remain to be answered to increase our understanding of SM toxicity and accelerate the development of vesicant therapeutics.

Sulfur mustard (SM, bis[2-chloroethyl]sulfide) is a highly reactive bifunctional alkylating agent that exerts a local toxic effect on skin, mucous membranes, eyes, and the respiratory tract.^1^ Several investigators have reported the signs and symptoms of the clinical and experimental acute effects of SM cutaneous injury. These findings are thoroughly reviewed by Papirmeister et al.^2^ and are summarized here. Clinical injury is characterized by an initial asymptomatic latent period of 1 to 12 hours that precedes lesion development. Low-dose SM exposure (vapor or liquid) produces a lesion that is mild and characterized by erythema, itching, and sensitivity to touch. With higher doses of SM, erythema develops more rapidly after latency, and is then followed by varying degrees of blistering and necrosis depending on dose. Histological observations of skin lesions show that, at early times (3–12 hours) in the erythematous stage of lesion development, signs of cytotoxicity appear first in basal keratinocytes.^3^–^6^ As the injury progresses, degenerative changes occur throughout the layers of the skin, but these changes are more prevalent in the basal keratinocytes of the epidermis. Past 12 hours, basal cell degeneration becomes more widespread. Foci of epidermal-dermal separation coalesce, leading to microscopic and then macroscopic blister formation,^3^,^5^ and inflammatory cell infiltrates can be observed in the epidermis and dermis.^3^–^8^ Visible blister formation occurs 12 to 48 hours after exposure and is maximal at 42 to 72 hours. The skin surface denudes between 6 and 9 days and scab formation occurs.^9^,^10^ Healing of SM cutaneous injuries is generally much slower than for other types of skin injury. Slow regeneration begins about a month after exposure and the healing process completes in about 2 to 3 months.^9^,^10^

The clinical and histopathological aspects of SM cutaneous exposure have been well studied, and, in fact, most of what we know in this regard has been complete since the mid-1940s. Only in the last 20 years has technology emerged to enable the study of the molecular and cellular response to SM. Given that the clinical presentation and histological findings of SM cutaneous lesions implicate an inflammatory component to this injury, mediators of inflammation were some of the earliest molecular signals investigated in SM injury. Several studies using a variety of animal skin and cultured cell models have identified numerous inflammatory mediators that are produced in response to SM exposure, including interleukin (IL)-1α, IL-1 β, IL-6, IL-8, granulocyte-monocyte colony-stimulating factor, GRO (growth factor and chemokine), leukotriene B_4_, monocyte chemoattractant (activating) protein-1, and tumor necrosis factor α (TNF-α).^11^–^23^ Many of the inflammatory mediators upregulated in SM exposure have well-characterized roles in the inflammatory phase of other types of dermal injury^22^ and likely perform similar functions in SM cutaneous injury. These mediators may also have functions that are unique to SM injury, but thus far only Qabar et al.^17^ have investigated this possibility. They showed that the expression of TNF-α altered keratinocyte sensitivity to SM-induced cell death.^17^ This observation is important as it suggests that limiting inflammation in SM exposure may limit injury. Anti-inflammatory compounds have been evaluated in SM cutaneous injury in mice, but have been only partially successful in reducing edema, epidermal necrosis, and subepidermal blistering.^18^,^19^ The inflammatory response in SM injury has not been fully characterized, and it is possible that inhibitors that more efficiently target critical mediators of the inflammatory response may prove beneficial. For example, expression of some mediators has been shown to precede erythema,^11^,^12^,^23^ and inhibiting these early events may attenuate the ensuing inflammatory response and limit injury. While inflammation is an important cascade of events in SM exposure that likely contributes to the observed injury, factors and events that are more directly implicated in tissue damage remain to be elucidated as well.

The involvement of serine and matrix metalloproteases in SM injury has been studied in the context of tissue injury downstream of the initial toxicity. Although SM alkylates numerous physiologically relevant molecules in cells and tissues,^24^ SM-induced DNA damage has been hypothesized to be the primary initiator of the cellular response that leads to the observed clinical injury.^25^–^30^ Papirmeister et al.^31^ were the first to propose a mechanism linking the initial SM-induced DNA damage and downstream protease activation resulting in blister formation. In their model, SM-induced DNA damage results in poly(ADP-ribose) polymerase (PARP) activation. Overactivation of PARP would lead to NAD^+;^ depletion and stimulation of the NADP^+;^-dependent hexosemonophosphate shunt, and would result in enhanced synthesis and release of proteases (specifically plasmin). They proposed that these proteases could be responsible for the development of subepidermal blisters by separating the basal cell layer from the basement membrane and allowing the accumulation of fluid in the resulting space. Since that time, numerous studies in various *in vitro* and *in vivo* systems have shown that increased protease expression and activity is associated with cutaneous SM injury.^12^,^32^–^45^ However, at this time, there are still no data demonstrating a causal link between SM-induced DNA damage, protease release due to metabolic disruption, and clinical injury. In addition, the pathways that regulate SM-induced protease expression and activity have not been identified and remain to be elucidated.

Although numerous clinical and molecular observations of SM injury have been reported, only a few studies have been able to establish a causative or direct relationship between observed molecular events and specific aspects of SM-induced injury. More recently though, experimental evidence reported by several investigators is beginning to define roles for NF-*κ* B, p53, p38, PARP, Fas, calcium, and calmodulin in the molecular mechanisms of SM-induced cell death, inflammation, and injury. This review focuses on these studies. Elucidating the molecular events following SM exposure is likely to identify the mechanisms of SM injury and the major regulators of the cellular response to SM. Knowledge of these regulators and their roles in the mechanisms of SM injury is necessary for the rational development of SM therapeutics. The goal for any therapeutic for SM injury would be not only to limit injury but also to do so without increasing survival of SM-exposed cells in a way that increases the risk of tumor formation, as SM is a known carcinogen.^46^ An understanding of the molecular mechanisms of SM-induced injury and cell death will be necessary to develop therapeutics that are effective at minimizing the acute injury and do not increase the risk of tumor formation.

## SIGNALING PATHWAYS ACTIVATED IN SM EXPOSURE

### NF-*κ* B, p38, and p53 signaling

The NF-*κ* B/Rel family of transcription factors mediates numerous cellular responses such as inflammation, apoptosis, proliferation, differentiation, and tumorigenesis.^47^ Several investigators have implicated NF-*κ* B as playing a role in inflammation in SM injury.^17^,^48^–^50^ However, all of these implications have been based on the observations that (1) SM-induced or SM analog–induced modulation of NF-*κ* B activity in gel shift assays correlates with protection or inflammatory events, and (2) NF-*κ* B exhibits proinflammatory activity in other systems.^51^,^52^ Collectively, this evidence certainly suggests a role for NF-*κ* B in SM injury; however, more definitive evidence that NF-*κ* B plays a role specifically in SM-induced cytokine production and inflammation has remained elusive. Another possible role for NF-*κ* B in SM injury is in cell death. Many of the studies that implicated NF-*κ* B in SM-induced inflammation also implicated NF-*κ* B in SM-induced cell death for similar reasons (ie, the observations that modulation of NF-*κ* B activity correlates with protective events and that NF-*κ* B has antiapoptotic activity in other systems).^53^–^55^ However, as with the role proposed for NF-*κ* B in SM-induced inflammation, definitive evidence of a role for NF-*κ* B in SM-induced cell death has not been reported.

Although the exact role of NF-*κ* B in SM-induced inflammation remains in question, recent data suggest that another signaling molecule, p38 MAP kinase, plays a critical role in SM-induced inflammatory cytokine production.^14^ p38 MAP kinase is related to the extracellular signal-regulated kinases and stress-activated protein kinases (SAPKs).^56^,^57^ Like the SAPKs, p38 can be activated by various stressors, including heat, UV irradiation, chemical shock, IL-1, and TNF-*α*. Once activated, p38 then phosphorylates and activates numerous molecular targets, including transcription factors that regulate expression of inflammatory cytokines. Dillman et al.^14^ investigated the role of p38 MAPK signaling in SM-exposed normal human epidermal keratinocytes (NHEKs) and observed that SM rapidly induced p38 phosphorylation (within 15 minutes) in a dose-dependent manner. The upstream kinase MKK3/MKK6 was also rapidly activated, consistent with the rapid phosphorylation of p38. Perhaps the most intriguing observation made in this study was that inhibiting p38 substantially and significantly reduced SM-induced inflammatory cytokine production. Treatment of NHEKs with SB203580, a p38 inhibitor, decreased the SM-induced production of IL-6, IL-8, and TNF-*α* by ∼90% and IL-1 *β* by ∼50%. While these findings do not rule out the involvement of NF-*κ* B in SM-induced inflammatory cytokine production, they do indicate that p38 may play a more critical role than NF-*κ* B in this regard.

**Figure 1:**
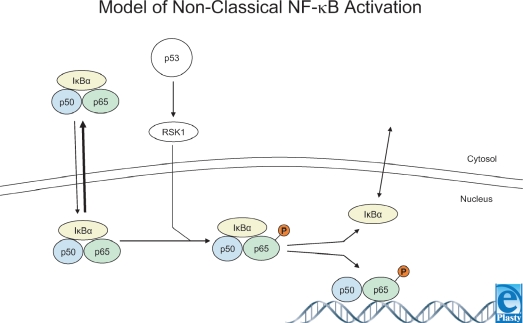
Model of nonclassical NF-*κ* B activation. A fraction of NF-*κ* B-I *κ* B*α* complexes shuttle into (and out of) the nucleus. p53 induces RSK1 activation. RSK1 translocates to the nucleus where it phosphorylates serine 536 of p65. This decreases the affinity of I*κ* B*α* for p65, and p50-p65 (NF-*κ* B) can then bind to NF-*κ* B responsive elements. p53 induces NF-*κ* B activation by an I*κ*-B kinase-independent mechanism involving phosphorylation of p65 by ribosomal S6 kinase 1. Figure adapted from Bohuslav et al.^59^ Used with permission.

While the role of NF-*κ* B in SM injury has been studied for several years, only recently has the mechanism of NF-*κ* B activation in SM injury been investigated. Minsavage and Dillman^58^ showed that activation of NF-*κ* B by SM was delayed and occurred 2 to 4 hours after exposure. Higher doses of SM did not induce a more rapid activation of NF-*κ* B. SM also induced phosphorylation of p90 ribosomal S6 kinase (p90RSK) 1 to 4 hours postexposure. In the classical scheme of NF-*κ* B activation, a stimulating agent (e.g. TNF-α) rapidly induces activation of NF-*κ* B.^47^ However, the observation reported by Minsavage and Dillman that NF-*κ* B and p90RSK activation is delayed in SM exposure is similar to the model of nonclassical NF-*κ* B activation reported by others.^59^ In this nonclassical model (Fig 1), NF-*κ* B activation is delayed for hours following stimulus, and activation is thought to result from p90RSK phosphorylation of Iκ B and/or p65.^59^–^61^ Minsavage et al.^58^ also showed that SM rapidly induced activation of p53 (within 15 minutes) as indicated by phosphorylation of amino acid residue Ser-15. p53 responds to a variety of genotoxic and nongenotoxic stressors, especially DNA damaging agents, and plays an important role in tumor suppression, cell cycle arrest, and apoptosis by transcription-dependent and independent mechanisms.^62^ Caffeic acid phenyl ester (CAPE) is a putative NF-*κ* B-specific inhibitor. However, Minsavage et al. demonstrated that CAPE inhibited not only both TNF-*α*-induced and SM-induced activation of NF-*κ* B but also the phosphorylation of p90RSK and p53. This suggests that CAPE may not be an NF-*κ* B-specific inhibitor. The observation that CAPE inhibited the activation of NF-*κ* B, p90RSK, and p53 leaves 3 possible explanations as to the mode of NF-*κ* B inhibition by CAPE. First, CAPE may have inhibited the nonclassical SM-induced activation of NF-*κ* B by directly acting on NF-*κ* B. Second, CAPE may have inhibited the nonclassical SM-induced activation of NF-*κ* B by inhibition of p90RSK. The third and perhaps most provocative possibility involves p53. CAPE inhibition of p53 activation occurred several hours before NF-*κ* B was observed to be activated and before NF-*κ* B inhibition by CAPE. Thus, p53 may be involved in SM-induced activation of NF-*κ* B, and inhibition of nonclassical SM-induced activation of NF-*κ* B by CAPE may occur via p53.

**Figure 2:**
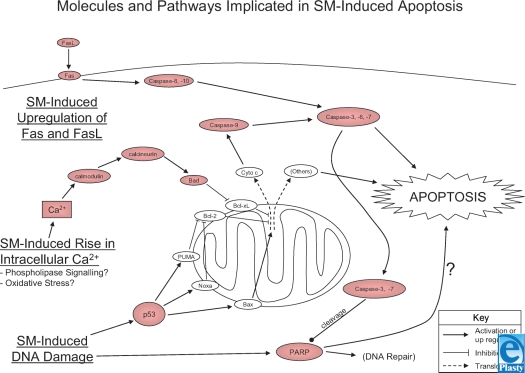
Molecules and pathways implicated in sulfur mustard (SM)-induced apoptosis. All molecules in red have been demonstrated to be activated or upregulated in SM exposure. Molecules in white have been demonstrated to play a fundamental role in apoptosis in many other systems but have not yet been directly implicated in SM injury. SM-induced DNA damage is believed to lead to p53 activation. SM exposure also induces a rise in the intracellular concentration of Ca^2+;^ though the mechanism of Ca^2+;^ release and precise insult leading to the release remain unclear. However both p53 and Ca^2+;^ signaling appear to lead to the activation of intrinsic mitochondrial pathways of apoptosis. While SM-induced DNA damage does induce PARP activation, the role of PARP in apoptosis may be cell-type dependent and the exact pathway remains to be demonstrated. SM exposure has also been demonstrated to lead to the upregulation of both Fas and FasL activating the extrinsic pathway of apoptosis. However, the mechanism(s) of upregulation of Fas and FasL in SM injury is unknown.

A role for p53 in SM cutaneous injury was first implicated by Smith et al.^63^ from the observation that p53 expression was increased in areas of cell death in SM-exposed pig skin. Stoppler et al.^64^ later demonstrated a role for p53 in SM-induced apoptosis of cultured keratinocytes. Cells were transformed with human papillomavirus-16 (HPV-16) E6 or E7 to inhibit or upregulate p53 expression, respectively. Keratinocytes expressing E6 were similar to untransformed cells in their sensitivity to SM-induced or TNF-*α*-induced apoptosis. However, E7 expressing cells were sensitized to SM-induced and TNF-*α*-induced apoptosis. Since E7 expression is accompanied by increased p53 levels, these findings implicate p53 in the apoptotic response to SM and TNF-*α* in these cells. These observations were confirmed by Rosenthal et al.,^65^ who also found that HPV-16 E7 sensitized keratinocytes to SM-induced apoptosis. In addition, these authors observed that SM-exposed keratinocytes expressing E7 also had higher levels of caspase-3 activity and PARP cleavage (PARP is a substrate of caspase-3) and suggested that the apoptotic response to SM is complex, involving multiple apoptotic signaling pathways, including p53, caspases, and PARP (Fig 2).

### PARP signaling

PARP uses NAD^+;^ as a substrate and catalyzes the addition and polymerization of ADP-ribose to a variety of nuclear proteins. PARP is induced by DNA strand breaks, and ADP ribosylation of nuclear proteins has a role in DNA repair and cell recovery following DNA damage.^66^ Papirmeister et al.^31^ showed that SM doses producing only mild injury resulted in a transient drop in NAD^+;^ levels that returned to normal level by 18 hours postexposure. However, with moderate and high vesicating doses of SM, PARP activation occurred to such a degree that cellular NAD^+;^ pools were depleted. From these observations, Papirmeister et al. proposed their mechanism of SM-induced PARP-mediated cell death leading to blister formation. Studies with other DNA-damaging agents supported this model of cell death by PARP-mediated depletion of NAD^+;^ induced by DNA damage.^67^–^69^ Although PARP can signal for cell death by depletion of intracellular pools of NAD^+;^ or ATP, this may not be the only mechanism by which PARP activation can signal for cell death. Recent studies indicate that the PARP product poly(ADP-ribose) (PAR) alone can signal for cell death, and degradation of PAR can protect against cell death.^70^,^71^ This may account, at least in part, for the observations of Mol et al.^72^ and Yourick et al.,^73^ who reported that reduction of skin damage by treatment with PARP inhibitors did not correlate with the NAD^+;^ content of the skin. Regardless of the mechanism of PARP-induced cell death (NAD^+;^ depletion and/or PAR synthesis), PARP inhibitors provided only a marginal degree of protection against cell death in culture and did not significantly ameliorate tissue damage.^72^,^73^ Other investigations that implicate PARP in SM-induced (or other agent-induced) cell death also report that protection by PARP inhibitors is limited.^74^–^76^ Furthermore, recent findings by Haince et al.^77^ bring into question the therapeutic value of PARP inhibitors for SM injury. They propose a model of DNA damage response in which PAR molecules attached to PARP recruit ataxia-telangiectasia mutated to sites of DNA damage. Their results showed that inhibition of PAR synthesis by PARP inhibitors and PARP knockout significantly reduced phosphorylation of proteins involved in the response to and repair of DNA damage, including p53, SMC1, and H2AX histone, suggesting that the ability of cells to respond to DNA damage is compromised in the absence of PARP activity. PARP inhibitors and PARP knockout did not protect cells, but actually sensitized cells to DNA damage–induced cell death. Their work suggests that PARP inhibitors compromise the ability of cells to respond to and repair DNA damage and may be detrimental as a therapeutic approach to treat injuries caused by DNA damaging agents such as SM. PARP has also been implicated in roles in SM injury other than mediating cell death. Hinshaw et al.^78^ proposed that the ATP-dependent disruption of microfilament architecture they observed following SM exposure was due to PARP-mediated NAD^+;^ and ATP depletion. Disrupted microfilament architecture altered keratinocyte morphology and increased the permeability of endothelial cell monolayers. They also noted that keratinocyte cell adherence was more sensitive to SM exposure than endothelial cell adherence. They suggested that these SM-induced PARP-mediated phenotypic changes might account for the capillary leakage and loss of cell adherence observed in SM cutaneous injury. In addition to altering microfilament architecture, PARP has been implicated in determining the mode of cell death in SM injury. Rosenthal et al.^65^ showed that PARP conferred a reduction in apoptotic markers in SM-exposed fibroblasts. However, this reduction of apoptotic markers was due to shifting the mode of death from apoptosis to necrosis by PARP.^79^ SM-exposed fibroblasts from PARP knockout mice expressed increased apoptotic markers relative to fibroblasts from normal mice. There was also a dose-dependent increase of necrotic markers in fibroblasts from normal mice but not in fibroblasts from PARP knockout mice. In contrast to fibroblasts, keratinocytes from both normal and PARP knockout mice exhibited markers of apoptosis when exposed to SM. Thus, PARP induced a shift in the mode of SM-induced cell death away from an apoptotic phenotype to a more necrotic phenotype in fibroblasts, but not in keratinocytes. Rosenthal et al. noted the work by Meier et al.^76^ in which PARP inhibitors reduced SM-induced necrosis in lymphocytes, but not markers of apoptosis, and suggested that PARP may induce a shift toward necrosis in SM-exposed lymphocytes similar to their observations in fibroblasts.

### Calcium signaling

Several studies implicate calmodulin and rises in intracellular Ca^2+;^ levels in SM toxicity,^80^–^84^ and these are perhaps the two most well-studied signaling molecules induced by SM exposure. Alterations in Ca^2+;^ homeostasis are thought to play a role in the cytotoxicity of some toxicants,^85^–^89^ and Ca^2+;^ and calmodulin have been shown to play a role in apoptosis.^83^,^90^–^92^ Two mechanisms have been proposed for the rise in Ca^2+;^ levels in apoptosis. The first mechanism involves protein kinase signaling pathways that lead to the activation of phospholipase C (PLC) and the generation of inositol triphosphate (IP_3_), which acts on Ca^2+;^ channels to release Ca^2+;^ from intracellular stores.^86^,^93^–^95^ The second mechanism involves oxidative stress in which reactive oxygen species generated by toxicant exposure react with Ca^2+;^ transport channels in the endoplasmic reticulum, mitochondria, and cell membrane. These reactions damage the Ca^2+;^ transport channels, which results in an influx of Ca^2+;^ into the cytosol.^93^,^96^ The mechanism(s) underlying the SM-induced rise in intracellular Ca^2+;^ has not been fully elucidated; however, results from studies thus far suggest two possible mechanisms that are similar to those described above. Ca^2+;^ levels have been shown to rise following oxidative or electrophilic stress because cellular protein thiol levels are depleted.^97^,^98^ Since SM has been shown to deplete glutathione levels,^99^ this is one possible mechanism for the rise in intracellular Ca^2+;^ in SM-exposed cells. The second possible mechanism involves phospholipase activation. There are no data demonstrating that SM induces the generation of IP_3_ and subsequent Ca^2+;^ release. However, SM exposure results in the activation of phospholipase D in cultured keratinocytes,^100^ and in other systems, phospholipase D is believed to elevate intracellular Ca^2+;^ levels by activating the Src/PLC*γ* pathway.^101^

The effect of SM on intracellular Ca^2+;^ levels has been studied in both fibroblasts^80^ and keratinocytes.^81^ In B77 fibroblasts, SM induced a dose-dependent rise in intracellular Ca^2+;^ levels that occurred within 5 to 10 minutes of exposure. SM also induced a rise in intracellular Ca^2+;^ levels in keratinocytes, but the response was more delayed. No rise in Ca^2+;^ was observed in the first 20 minutes after exposure, but a rise in Ca^2+;^ was observed at later time points of 3 to 24 hours. SM also affected Ca^2+;^ signaling in response to extracellular signals in both cell types, but with different kinetics and with different dynamics. In fibroblasts, SM completely inhibited Ca^2+;^ signaling responses to serum within about 40 minutes of exposure. In contrast, the rise of intracellular Ca^2+;^ in response to histamine and ATP was attenuated in SM-exposed keratinocytes in a dose-dependent and time-dependent manner, but was not completely inhibited. However, comparison of all the data from the two studies is difficult because of differences in the assays. A slightly higher dose of SM (1 mM) was used with fibroblasts than the highest dose of SM (0.8 mM) used with keratinocytes. This may account for some of the differences in the responses, but these doses are similar, and inherent differences in the response of fibroblasts versus keratinocytes would seem to be a more likely explanation. Comparisons of the responses with extracellular signals are much more difficult because of the very different extracellular signals used (serum vs ATP and histamine). Additional studies, using identical SM doses, extracellular signals, and culture conditions, are necessary to establish whether differences in the responses are inherent to the two cell types. In spite of the differences in the assays, some important implications can be drawn from these studies. First, both studies show that SM affected the ability of cells to respond to extracellular signals. This implies that SM-induced cell death may result, at least in part, from an inability to respond to survival and growth signals present in the extracellular milieu. Second, both studies show that the rise in intracellular Ca^2+;^ was moderate (<2-fold). Both authors suggested that the modest increases in intracellular Ca^2+;^ levels induced by SM were more likely to cause aberrant physiology rather than toxicity, since intracellular Ca^2+;^-mediated toxicity requires much higher levels.^80^,^81^

The suggestion by Hua et al.^80^ and Mol and Smith^81^ that SM-induced changes in Ca^2+;^ levels alter cell physiology but do not cause cellular toxicity has been supported by the work of Rosenthal et al.^65^,^83^ They reported that keratinocytes exposed to SM expressed markers of terminal differentiation (induction of K1/K10, crosslinking of involucrin, suppression of fibronectin) and apoptosis (induction of p53, suppression of Bcl-2, activation of caspase-3, PARP cleavage, induction of DNA fragmentation).^65^,^83^ The Ca^2+;^ ionophore BAPTA-AM, the calmodulin inhibitor W7, and calmodulin antisense RNA suppressed these markers. This suggests a role for Ca^2+;^ and calmodulin in the SM-induced terminal differentiation and apoptotic processes. These observations are consistent with other studies on the role of Ca^2+;^ in terminal differentiation of keratinocytes,^102^–^107^ and the role of Ca^2+;^ and calmodulin in apoptosis.^92^,^108^ Rosenthal et al.^83^ suggested that the involvement of Ca^2+;^, calmodulin, p53, Bcl-2, PARP, and caspase-3 indicates that a complex signaling network is involved in SM-induced apoptosis. These findings also demonstrate that two potential mechanisms are involved in SM-induced keratinocyte death: apoptosis and terminal differentiation.

More recent work by Simbulan-Rosenthal et al.^84^ has further elucidated the role of calmodulin in SM-induced apoptosis. Calmodulin antisense RNA was shown to suppress the SM-induced activation of caspase−3, −6, −7, −8, and −9 as well as the proteolytic processing of PARP. However, calmodulin antisense RNA did not suppress caspase-3 activation induced by a Fas antibody agonist, suggesting that calmodulin is acting on other apoptotic pathways in SM exposure. Furthermore, calmodulin and calcineurin inhibitors both suppressed SM-induced caspase-3 activity, but calmodulin-dependent protein kinase II (CaM-kinase II) inhibitors did not. Calmodulin has been shown to modulate apoptosis via CaM-kinase II^109^,^110^ and via calcineurin.^92^ However, these results suggest that CaM-kinase II does not play a role in SM-induced apoptosis. Calcineurin has been shown to dephosphorylate and activate BAD, a proapoptotic protein that, when dephosphorylated, interacts with the antiapoptotic proteins Bcl-2 or Bcl-XL to induce apoptosis.^111^–^113^ Rosenthal et al. showed that BAD was completely dephosphorylated 24 hours after SM exposure, and inhibition of calmodulin by antisense RNA or W13 (a calmodulin inhibitor) inhibited the SM-induced dephosphorylation of BAD and attenuated nuclear fragmentation. Rosenthal et al. also suggest that SM induces apoptosis in keratinocytes by a calmodulin-BAD mitochondrial pathway. However, they further suggest that both death receptor (Fas) and mitochondrial apoptotic pathways are involved in SM-induced apoptosis, given that they also observed processing and activation of caspase-8 and −9, which were only partially blocked by calmodulin 1 antisense RNA. The involvement of both Fas-mediated and mitochondrial pathways of apoptosis in the response to SM was also reported in earlier work by this group.^114^ The intrinsic and extrinsic pathways of apoptosis implicated in SM injury are summarized in Figure 2.

### Fas signaling

In their earlier study implicating Fas and mitochondrial apoptotic pathways in SM injury, Rosenthal et al.^114^ investigated Fas apoptotic signaling. SM induced the upregulation of Fas and FasL in keratinocytes as well as processing of procaspase-3, −7, −8 and −9. SM-induced apoptosis and caspase-3 activity was also diminished by Fas-blocking antibody. The observation of caspase-8 and −9 activation suggests that both death receptor and mitochondrial apoptotic pathways are involved in SM-induced cell death. Also in this study, they further investigated the role of Fas using keratinocytes from Fas knockout mouse pups and keratinocytes transformed by HPV-16 E6/E7 that stably expressed a dominant-negative, Fas-activated death domain (FADD-DN). FADD-DN suppressed SM-induced DNA fragmentation as well as caspase-3, −6, and −8 processing and/or activity. FADD-DN was also shown to partially diminish SM-induced vesication of grafts on nude mice. Keratinocytes from Fas knockout pups showed a reduction in SM-induced caspase-3 activation and DNA strand breaks relative to controls. Rosenthal et al. noted that markers of apoptosis were only diminished by FADD-DN and Fas knockout, consistent with the involvement of other pathways (mitochondrial pathways) in SM-induced apoptosis.

## FUTURE DIRECTIONS

Progress has been made in elucidating the signaling pathways involved in SM toxicity. This is especially true of the investigations on the role of Ca^2+;^ and calmodulin in SM injury and some of the more recent studies with other signaling molecules. However, the proposed roles for many of the molecular events in SM injury remain mostly theoretical, as causative or direct relationships between them and specific aspects of SM injury have yet to be experimentally established. This is critical to the rational development of vesicant therapeutics. Perhaps, a foundational key connection that needs to be elucidated is the relationship between SM-induced DNA damage and the ensuing signaling events that lead to clinical injury. Also, while defining the roles of downstream signaling molecules in SM injury is obviously important, determining how crosstalk between these pathways affects the cellular response and how SM exposure dose affects all of these responses and relationships is likely to be very important as well. For example, is there a direct causative link between DNA damage and inflammation or are these pathways activated in parallel by distinctly different mechanisms induced by SM exposure? What is the relationship between NF-*κ* B and p38 in the production of inflammatory cytokines? What are the transcription factors downstream of p38 that activate expression of inflammatory cytokines? Although the connection between DNA damage and cell death is well studied with model genotoxic agents (eg, UV irradiation), this connection has not been completely established for SM toxicity. What is the relationship between p53, NF-*κ* B, and apoptosis? What are the signals that activate the p53 response? What are the downstream targets of p53 and NF-*κ* B and how are they related to the process of SM-induced apoptosis? Is it possible to allow injured cells to die and yet decrease the robust inflammatory response? Finally, what is the relationship between each of these signaling pathways and the formation of large bolus blisters that are the hallmark of cutaneous SM exposure? Answers to these key questions are vital to increasing our understanding of SM toxicity and accelerating the development of vesicant therapeutics.

## References

[1] Medema J (1986). Mustard gas: the science of H. Nucl Biol Chem Def Technol Int.

[2] Papirmeister B, Feister A, Robinson S, Ford R (1991). Medical Defense Against Mustard Gas: Toxic Mechanisms and Pharmacological Implications.

[3] Henriques FC, Moritz AR, Breyfogle HS, Patterson LA (1943). Washington, DC: Division 9, National Defense Research Committee of the Office of Scientific Research and Development. The Mechanism of Cutaneous Injury by Mustard Gas. An Experimental Study Using Mustard Prepared with Radioactive Sulfur.

[4] Ginzler AM, Davis MI (1943). Edgewood Arsenal, Md: US Army Medical Research Laboratory. The Pathology of Mustard Burns of Human Skin.

[5] Pappenheimer AM (1926). Pathological actions of war gases. In: Lynch C, ed. The Medical Department of the United States Army in the World War. Vol 14: Medical Aspects of Gas Warfare.

[6] Willems KL (1989). Clinical management of mustard gas casualties. Ann Med Milit (Belg).

[7] Mehzad M (1988). Pathological study of skin lesions in chemical casualties abstract. Abstract presented at: Abstracts of the First International Medical Congress on Chemical Warfare Agents in Iran; Mashhad University of Medical Sciences, Mashhad, Iran.

[8] Warthin AS, Weller CV (1919). The Medical Aspects of Mustard Gas Poisoning.

[9] Renshaw B (1946). Mechanisms in production of cutaneous injuries by sulfur and nitrogen mustards. In: *Chemical Warfare Agents, and Related Chemical Problems*. Washington, DC: Division 9, National Defense Research Committee of the Office of Scientific Research and Development.

[10] Nagy SM, Golumbic C, Stein WH, Fruton JS, Bergmann M (1946). The penetration of vesicant vapors into human skin. J Gen Physiol.

[11] Sabourin CL, Petrali JP, Casillas RP (2000). Alterations in inflammatory cytokine gene expression in sulfur mustard-exposed mouse skin. J Biochem Mol Toxicol.

[12] Sabourin CL, Danne MM, Buxton KL, Casillas RP, Schlager JJ (2002). Cytokine, chemokine, and matrix metalloproteinase response after sulfur mustard injury to weanling pig skin. J Biochem Mol Toxicol.

[13] Arroyo CM, Schafer RJ, Kurt EM, Broomfield CA, Carmichael AJ (1999). Response of normal human keratinocytes to sulfur mustard (HD): cytokine release using a non-enzymatic detachment procedure. Hum Exp Toxicol.

[14] Dillman JF, McGary KL, Schlager JJ (2004). An inhibitor of p38 MAP kinase downregulates cytokine release induced by sulfur mustard exposure in human epidermal keratinocytes. Toxicol In Vitro.

[15] Lardot C, Dubois V, Lison D (1999). Sulfur mustard upregulates the expression of interleukin-8 in cultured human keratinocytes. Toxicol Lett.

[16] Arroyo CM, Broomfield CA, Hackley BE (2001). The role of interleukin-6 (IL-6) in human sulfur mustard (HD) toxicology. Int J Toxicol.

[17] Qabar A, Nelson M, Guzman J (2005). Modulation of sulfur mustard induced cell death in human epidermal keratinocytes using IL-10 and TNF-alpha. J Biochem Mol Toxicol.

[18] Casillas RP, Kiser RC, Truxall JA (2000). Therapeutic approaches to dermatotoxicity by sulfur mustard; part I: modulation of sulfur mustard-induced cutaneous injury in the mouse ear vesicant model. J Appl Toxicol.

[19] Babin MC, Ricketts K, Skvorak JP (2000). Systemic administration of candidate antivesicants to protect against topically applied sulfur mustard in the mouse ear vesicant model (MEVM). J Appl Toxicol.

[20] Ricketts KM, Santai CT, France JA (2000). Inflammatory cytokine response in sulfur mustard-exposed mouse skin. J Appl Toxicol.

[21] Blank JA, Lane LA, Menton RG, Casillas RP (2000). Procedure for assessing myeloperoxidase and inflammatory mediator responses in hairless mouse skin. J Appl Toxicol.

[22] Werner S, Grose R (2003). Regulation of wound healing by growth factors and cytokines. Physiol Rev.

[23] Tsuruta J, Sugisaki K, Dannenberg AM (1996). The cytokines NAP-1 (IL-8), MCP-1, IL-1 beta, and GRO in rabbit inflammatory skin lesions produced by the chemical irritant sulfur mustard. Inflammation.

[24] Stein WH (1946). Chemical reaction of sulfur and nitrogen mustards. In: *Chemical Warfare Agents, and Related Chemical Problems–Parts III–VI*. Washington, DC: Division 9, National Defense Research Committee of the Office of Scientific Research and Development.

[25] Herriott RM, Price WH (1948). The formation of bacterial viruses in bacteria rendered non-viable by mustard gas. J Gen. Physiol.

[26] Fox M, Scott D (1980). The genetic toxicity of nitrogen and sulphur mustard. Mutat Res.

[27] Papirmeister B, Davison CL (1964). Elimination of sulfur mustard-induced products from DNA of *Escherichia coli*. Biochem Biophys Res Commun.

[28] Lawley PD, Brookes P (1965). Molecular mechanism of the cytotoxic action of difunctional alkylating agents and of resistance to this action. Nature.

[29] Lawley PD, Brookes P (1968). Cytotoxicity of alkylating agents towards sensitive and resistant strains of *Escherichia coli* in relation to extent and mode of alkylation of cellular macromolecules and repair of alkylation lesions in deoxyribonucleic acids. Biochem J.

[30] Papirmeister B, Westling AW, Schroer J (1969). Mustard: The Relevance of DNA Damage to the Development of the Skin Lesion.

[31] Papirmeister B, Gross CL, Meier HL, Petrali JP, Johnson JB (1985). Molecular basis for mustard-induced vesication. Fundam Appl Toxicol.

[32] Harada S, Dannenberg AM, Vogt RF (1987). Inflammatory mediators and modulators released in organ culture from rabbit skin lesions produced in vivo by sulfur mustard; part III: electrophoretic protein fractions, trypsin-inhibitory capacity, alpha 1-proteinase inhibitor, and alpha 1- and alpha 2-macroglobulin proteinase inhibitors of culture fluids and serum. Am J Pathol.

[33] Kajiki A, Higuchi K, Nakamura M (1988). Sources of extracellular lysosomal enzymes released in organ-culture by developing and healing inflammatory lesions. J Leukoc Biol.

[34] Higuchi K, Kajiki A, Nakamura M (1988). Proteases released in organ culture by acute dermal inflammatory lesions produced in vivo in rabbit skin by sulfur mustard: hydrolysis of synthetic peptide substrates for trypsin-like and chymotrypsin-like enzymes. Inflammation.

[35] Woessner JF, Dannenberg AM, Pula PJ (1990). Extracellular collagenase, proteoglycanase and products of their activity, released in organ culture by intact dermal inflammatory lesions produced by sulfur mustard. J Invest Dermatol.

[36] Rikimaru T, Nakamura M, Yano T (1991). Mediators, initiating the inflammatory response, released in organ culture by full-thickness human skin explants exposed to the irritant, sulfur mustard. J Invest Dermatol.

[37] Cowan FM, Yourick JJ, Hurst CG, Broomfield CA, Smith WJ (1993). Sulfur mustard-increased proteolysis following in vitro and in vivo exposures. Cell Biol Toxicol.

[38] Lindsay CD, Rice P (1995). Changes in connective tissue macromolecular components of Yucatan mini-pig skin following application of sulphur mustard vapour. Hum Exp Toxicol.

[39] Lindsay CD, Rice P (1996). Assessment of the biochemical effects of percutaneous exposure of sulphur mustard in an in vitro human skin system. Hum Exp Toxicol.

[40] Lindsay CD, Upshall DG (1995). The generation of a human dermal equivalent to assess the potential contribution of human dermal fibroblasts to the sulphur mustard-induced vesication response. Hum Exp Toxicol.

[41] Chakrabarti AK, Ray P, Broomfield CA, Ray R (1998). Purification and characterization of protease activated by sulfur mustard in normal human epidermal keratinocytes. Biochem Pharmacol.

[42] Powers JC, Kam CM, Ricketts KM, Casillas RP (2000). Cutaneous protease activity in the mouse ear vesicant model. J Appl Toxicol.

[43] Cowan FM, Broomfield CA, Smith WJ (2002). Suppression of sulfur mustard-increased IL-8 in human keratinocyte cell cultures by serine protease inhibitors: implications for toxicity and medical countermeasures. Cell Biol Toxicol.

[44] Wormser U, Brodsky B, Reich R (2002). Topical treatment with povidone iodine reduces nitrogen mustard-induced skin collagenolytic activity. Arch Toxicol.

[45] Shakarjian MP, Bhatt P, Gordon MK (2006). Preferential expression of matrix metalloproteinase-9 in mouse skin after sulfur mustard exposure. J Appl Toxicol.

[46] (2002). Program NT. Mustard gas. 11th Rep Carcinog.

[47] Chen LF, Greene WC (2004). Shaping the nuclear action of NF-κ B. Nat Rev Mol Cell Biol.

[48] Atkins KB, Lodhi IJ, Hurley LL, Hinshaw DB (2000). N-Acetylcysteine and endothelial cell injury by sulfur mustard. J Appl Toxicol.

[49] Chatterjee D, Mukherjee S, Smith MG, Das SK (2003). Signal transduction events in lung injury induced by 2-chloroethyl ethyl sulfide, a mustard analog. J Biochem Mol Toxicol.

[50] Das SK, Mukherjee S, Smith MG, Chatterjee D (2003). Prophylactic protection by N-acetylcysteine against the pulmonary injury induced by 2-chloroethyl ethyl sulfide, a mustard analogue. J Biochem Mol Toxicol.

[51] Baeuerle PA, Henkel T (1994). Function and activation of NF-kappa B in the immune system. Annu Rev Immunol.

[52] Christman JW, Sadikot RT, Blackwell TS (2000). The role of nuclear factor-kappa B in pulmonary diseases. Chest.

[53] Beg AA, Baltimore D (1996). An essential role for NF-kappaB in preventing TNF-alpha-induced cell death. Science.

[54] Xu Y, Bialik S, Jones BE (1998). NF-κ B inactivation converts a hepatocyte cell line TNF-alpha response from proliferation to apoptosis. Am J Physiol.

[55] Plumpe J, Malek NP, Bock CT (2000). NF-κ B determines between apoptosis and proliferation in hepatocytes during liver regeneration. Am J Physiol Gastrointest Liver Physiol.

[56] Chang L, Karin M (2001). Mammalian MAP kinase signalling cascades. Nature.

[57] Lee JC, Kassis S, Kumar S, Badger A, Adams JL (1999). p38 mitogen-activated protein kinase inhibitors–mechanisms and therapeutic potentials. Pharmacol Ther.

[58] Minsavage GD, Dillman JF (2007). Bifunctional alkylating agent-induced p53 and nonclassical nuclear factor κ B responses and cell death are altered by caffeic acid phenethyl ester: a potential role for antioxidant/electrophilic response-element signaling.. J Pharmacol Exp Ther.

[59] Bohuslav J, Chen LF, Kwon H, Mu Y, Greene WC (2004). p53 induces NF-kappaB activation by an Iκ B kinase-independent mechanism involving phosphorylation of p65 by ribosomal S6 kinase 1. J Biol Chem.

[60] Ghoda L, Lin X, Greene WC (1997). The 90-kDa ribosomal S6 kinase (pp90rsk) phosphorylates the N-terminal regulatory domain of Iκ Balpha and stimulates its degradation in vitro. J Biol Chem.

[61] Schouten GJ, Vertegaal AC, Whiteside ST (1997). Iκ B alpha is a target for the mitogen-activated 90 kDa ribosomal S6 kinase. EMBO J.

[62] Gomez-Lazaro M, Fernandez-Gomez FJ, Jordan J (2004). p53: twenty five years understanding the mechanism of genome protection. J Physiol Biochem.

[63] Smith KJ, Graham JS, Hamilton TA (1997). Immunohistochemical studies of basement membrane proteins and proliferation and apoptosis markers in sulfur mustard induced cutaneous lesions in weanling pigs. J Dermatol Sci.

[64] Stoppler H, Stoppler MC, Johnson E (1998). The E7 protein of human papillomavirus type 16 sensitizes primary human keratinocytes to apoptosis. Oncogene.

[65] Rosenthal DS, Simbulan-Rosenthal CM, Iyer S (2000). Calmodulin poly(ADP-ribose)polymerase and p53 are targets for modulating the effects of sulfur mustard. J Appl Toxicol.

[66] Grube K, Burkle A (1992). Poly(ADP-ribose) polymerase activity in mononuclear leukocytes of 13 mammalian species correlates with species-specific life span. Proc Natl Acad Sci USA.

[67] Wielckens K, Schmidt A, George E, Bredehorst R, Hilz H (1982). DNA fragmentation and NAD depletion. Their relation to the turnover of endogenous mono(ADP-ribosyl) and poly(ADP-ribosyl) proteins. J Biol Chem.

[68] Alvarez-Gonzalez R, Eichenberger R, Althaus FR (1986). Poly(ADP-ribose) biosynthesis and suicidal NAD+; depletion following carcinogen exposure of mammalian cells. Biochem Biophys Res Commun.

[69] Berger NA, Sims JL, Catino DM, Berger SJ (1983). Poly(ADP-ribose) polymerase mediates the suicide response to massive DNA damage: studies in normal and DNA-repair defective cells. Princess Takamatsu Symp.

[70] Yu SW, Andrabi SA, Wang H (2006). Apoptosis-inducing factor mediates poly(ADP-ribose) (PAR) polymer-induced cell death. Proc Natl Acad Sci USA.

[71] Andrabi SA, Kim NS, Yu SW (2006). Poly(ADP-ribose) (PAR) polymer is a death signal. Proc Natl Acad Sci USA.

[72] Mol ME, de Vries R, Kluivers AW (1991). Effects of nicotinamide on biochemical changes and microblistering induced by sulfur mustard in human skin organ cultures. Toxicol Appl Pharmacol.

[73] Yourick JJ, Clark CR, Mitcheltree LW (1991). Niacinamide pretreatment reduces microvesicle formation in hairless guinea pigs cutaneously exposed to sulfur mustard. Fundam Appl Toxicol.

[74] Meier HL, Johnson JB (1992). The determination and prevention of cytotoxic effects induced in human lymphocytes by the alkylating agent 2,2′;-dichlorodiethyl sulfide (sulfur mustard HD). Toxicol Appl Pharmacol.

[75] (1996). Meier HL The time-dependent effect of 2,2′-dichlorodiethyl sulfide (sulfur mustard HD 1,1′)-thiobis [2-chloroethane]) on the lymphocyte viability and the kinetics of protection by poly(ADP-ribose) polymerase inhibitors. Cell Biol Toxicol.

[76] Meier HL, Millard CB (1998). Alterations in human lymphocyte DNA caused by sulfur mustard can be mitigated by selective inhibitors of poly(ADP-ribose) polymerase. Biochim Biophys Acta.

[77] Haince JF, Kozlov S, Dawson VL (2007). ATM signaling network is modulated by a novel PAR-dependent pathway in the early response to DNA damaging agents. J Biol Chem.

[78] Hinshaw DB, Lodhi IJ, Hurley LL, Atkins KB, Dabrowska MI (1999). Activation of poly [ADP-ribose] polymerase in endothelial cells and keratinocytes: role in an in vitro model of sulfur mustard-mediated vesication. Toxicol Appl Pharmacol.

[79] Rosenthal DS, Simbulan-Rosenthal CM, Liu WF (2001). PARP determines the mode of cell death in skin fibroblasts, but not keratinocytes, exposed to sulfur mustard. J Invest Dermatol.

[80] Hua A, Daniel R, Jasseron MP, Thiriot C (1993). Early cytotoxic effects induced by bis-chloroethyl sulphide (sulphur mustard) [Ca^2+^]_i_ rise and time-dependent inhibition of B77 fibroblast serum response. J Appl Toxicol.

[81] Mol MA, Smith WJ (1996). Ca^2+^ homeostasis and Ca^2+^ signalling in sulphur mustard-exposed normal human epidermal keratinocytes. Chem Biol Interact.

[82] Ray R, Legere RH, Majerus BJ, Petrali JP (1995). Sulfur mustard-induced increase in intracellular free calcium level and arachidonic acid release from cell membrane. Toxicol Appl Pharmacol.

[83] Rosenthal DS, Simbulan-Rosenthal CM, Iyer S (1998). Sulfur mustard induces markers of terminal differentiation and apoptosis in keratinocytes via a Ca^2+^-calmodulin and caspase-dependent pathway. J Invest Dermatol.

[84] Simbulan-Rosenthal CM, Ray R, Benton B (2006). Calmodulin mediates sulfur mustard toxicity in human keratinocytes. Toxicology.

[85] Nicotera P, Bellomo G, Orrenius S (1992). Calcium-mediated mechanisms in chemically induced cell death. Annu Rev Pharmacol Toxicol.

[86] Trump BF, Berezesky IK, Smith MW, Phelps PC, Elliget KA (1989). The relationship between cellular ion deregulation and acute and chronic toxicity. Toxicol Appl Pharmacol.

[87] Reed DJ (1990). Review of the current status of calcium and thiols in cellular injury. Chem Res Toxicol.

[88] Pounds JG (1990). The role of cell calcium in current approaches to toxicology. Environ Health Perspect.

[89] Corcoran GB, Ray SD (1992). The role of the nucleus and other compartments in toxic cell death produced by alkylating hepatotoxicants. Toxicol Appl Pharmacol.

[90] Sasaki M, Uchiyama J, Ishikawa H (1996). Induction of apoptosis by calmodulin-dependent intracellular Ca^2+^ elevation in CD4^+^ cells expressing gp 160 of HIV. Virology.

[91] Pan Z, Radding W, Zhou W (1996). Role of calmodulin in HIV-potentiated Fas-mediated apoptosis. Am J Pathol.

[92] Shi YF, Sahai BM, Green DR (1989). Cyclosporin A inhibits activation-induced cell death in T-cell hybridomas and thymocytes. Nature.

[93] Orrenius S, McConkey DJ, Bellomo G, Nicotera P (1989). Role of Ca^2+^ in toxic cell killing. Trends Pharmacol Sci.

[94] Takata M, Homma Y, Kurosaki T (1995). Requirement of phospholipase C-gamma 2 activation in surface immunoglobulin M-induced B cell apoptosis. J Exp Med.

[95] Silvennoinen O, Nishigaki H, Kitanaka A (1996). CD38 signal transduction in human B cell precursors. Rapid induction of tyrosine phosphorylation, activation of syk tyrosine kinase, and phosphorylation of phospholipase C-gamma and phosphatidylinositol 3-kinase. J Immunol.

[96] Boobis AR, Fawthrop DJ, Davies DS (1989). Mechanisms of cell death. Trends Pharmacol Sci.

[97] (1990). Reed DJ Glutathione: toxicological implications. Annu Rev Pharmacol Toxicol.

[98] Nicotera P, Bellomo G, Orrenius S (1990). The role of Ca^2+^ cell killing. Chem Res Toxicol.

[99] Gentilhomme E, Neveux Y, Hua A (1992). Action of bis(betachloroethyl)sulphide (BCES) on human epidermis reconstituted in culture: morphological alterations and biochemical depletion of glutathione. Toxicol In Vitro.

[100] Lefkowitz LJ, Smith WJ (2002). Sulfur mustard-induced arachidonic acid release is mediated by phospholipase D in human keratinocytes. Biochem Biophys Res Commun.

[101] Sato K, Fukami Y, Stith BJ (2006). Signal transduction pathways leading to Ca^2+^ release in a vertebrate model system: lessons from *Xenopus* eggs. Semin Cell Dev Biol.

[102] Rosenthal DS, Steinert PM, Chung S (1991). A human epidermal differentiation-specific keratin gene is regulated by calcium but not negative modulators of differentiation in transgenic mouse keratinocytes. Cell Growth Differ.

[103] Stanley JR, Yuspa SH (1983). Specific epidermal protein markers are modulated during calcium-induced terminal differentiation. J Cell Biol.

[104] Hennings H, Michael D, Cheng C (1980). Calcium regulation of growth and differentiation of mouse epidermal cells in culture. Cell.

[105] Li L, Tucker RW, Hennings H, Yuspa SH (1995). Chelation of intracellular Ca^2+^ inhibits murine keratinocyte differentiation in vitro. J Cell Physiol.

[106] Kruszewski FH, Hennings H, Tucker RW, Yuspa SH (1991). Differences in the regulation of intracellular calcium in normal and neoplastic keratinocytes are not caused by ras gene mutations. Cancer Res.

[107] Kruszewski FH, Hennings H, Yuspa SH, Tucker RW (1991). Regulation of intracellular free calcium in normal murine keratinocytes. Am J Physiol.

[108] Kaiser N, Edelman IS (1977). Calcium dependence of glucocorticoid-induced lymphocytolysis. Proc Natl Acad Sci USA.

[109] Wright SC, Schellenberger U, Wang H, Wang Y, Kinder DH (1998). Chemotherapeutic drug activation of the AP24 protease in apoptosis: requirement for caspase 3-like-proteases. Biochem Biophys Res Commun.

[110] Wright SC, Wang H, Wei QS, Kinder DH, Larrick JW (1998). Bcl-2-mediated resistance to apoptosis is associated with glutathione-induced inhibition of AP24 activation of nuclear DNA fragmentation. Cancer Res.

[111] Wang HG, Pathan N, Ethell IM (1999). Ca^2+^-induced apoptosis through calcineurin dephosphorylation of BAD. Science.

[112] Yang E, Zha J, Jockel J (1995). Bad, a heterodimeric partner for Bcl-XL and Bcl-2, displaces Bax and promotes cell death. Cell.

[113] Zha J, Harada H, Osipov K (1997). BH3 domain of BAD is required for heterodimerization with BCL-XL and pro-apoptotic activity. J Biol Chem.

[114] Rosenthal DS, Velena A, Chou FP (2003). Expression of dominant-negative Fas-associated death domain blocks human keratinocyte apoptosis and vesication induced by sulfur mustard. J Biol Chem.

